# Central fatigue and nycthemeral change of serum tryptophan and serotonin in the athletic horse

**DOI:** 10.1186/1740-3391-3-6

**Published:** 2005-04-28

**Authors:** Giuseppe Piccione, Anna Assenza, Francesco Fazio, Maurizio Percipalle, Giovanni Caola

**Affiliations:** 1 Dipartimento di Morfologia, Biochimica, Fisiologia e Produzioni Animali, Facoltà di Medicina Veterinaria, Università degli Studi di Messina, 98168 Messina, Italy

## Abstract

**Background:**

The serotonergic system is associated with numerous brain functions, including the resetting of the mammalian circadian clock. The synthesis and metabolism of 5-HT in the brain increases in response to exercise and is correlated with high levels of blood-borne tryptophan (TRP). The present investigation was aimed at testing the existence of a daily rhythm of TRP and 5-HT in the blood of athletic horses.

**Methods:**

Blood samples from 5 Thoroughbred mares were collected at 4-hour intervals for 48 hours (starting at 08:00 hours on day 1 and finishing at 4:00 on day 2) via an intravenous cannula inserted into the jugular vein. Tryptophan and serotonin concentrations were assessed by HPLC. Data analysis was conducted by one-way repeated measures analysis of variance (ANOVA) and by the single cosinor method.

**Results:**

ANOVA showed a highly significant influence of time both on tryptophan and on serotonin, in all horses, on either day, with p values < 0.0001. Cosinor analysis identified the periodic parameters and their acrophases (expressed in hours) during the 2 days of monitoring. Both parameters studied showed evening acrophases.

**Conclusion:**

The results showed that serotonin and tryptophan blood levels undergo nycthemeral variation with typical evening acrophases. These results enhance the understanding of the athlete horse's chronoperformance and facilitate the establishment of training programs that take into account the nycthemeral pattern of aminoacids deeply involved in the onset of central fatigue.

## Background

Fatigue is an important factor affecting exercise and sporting performances. It is defined physiologically as the inability to maintain power output, [14] and the organism uses it as a defence mechanism to avoid irreversible damage due to excessive exertion. Fatigue is a complex multifactorial element with peripheral and central components. Central fatigue develops in the central nervous system and involves brain serotonin (5-HT) level [15]. The serotonergic system is associated with numerous brain functions that can positively or negatively affect endurance [6]. Accordingly, the synthesis and metabolism of 5-HT in the brain increases in response to exercise [4]. Furthermore, the rise of brain serotonin concentration is associated with markers of central fatigue such as decreased motivation, lethargy, tiredness and loss of motor coordination [6].

Increase of 5-HT synthesis in the brain is correlated with high levels of blood-borne tryptophan (TRP), the amino acid precursor to serotonin. The rate limiting step in the synthesis of 5-HT is the transport of TRP across the blood-brain barrier into the brain [9].

Many behavioral and physiological processes display 24-hour rhythms that are controlled by the circadian clock mechanism. The internal circadian clock is a molecular time-keeping system that generates a biological rhythm, regulating diverse physiological processes [18]. Serotonin (5-HT) is an important neurotransmitter and plays important roles in many physiological functions, including the operation of the mammalian circadian clock [12,17]. 5-HT is synthesized from the amino acid tryptophan hydroxylase (TPH) and aromatic L-amino acid decarboxylase [2,11]. 5-HT is a metabolic precursor of melatonin in the pineal gland and is believed to be involved in the control of sleep and in clock resetting [10].

In view of the above, and taking into account that the modulation of the physiological onset of fatigue acts positively on exercise adaptation, a good athlete's goal is to delay the occurrence of fatigue in order to maintain high performance standards. This can be achieved through a specific and continuous training programme [3]. Consequently, training and sporting activity in the horse should be accomplished in the predictably favourable phase of the day.

The present investigation was aimed at testing the existence of a daily rhythm of TRP and 5-HT in the plasma of horses. For this purpose, plasma samples were collected across 48 h from horses exposed to natural photoperiodic conditions and subjected to regular feeding and training schedules.

## Materials and methods

Five Thoroughbred mares, 8 years old, were used. For 30 days prior to the study, the animals underwent the same pattern of daily activity. They were housed in individual stalls under a natural photoperiod (sunrise at 06:06, sunset at 18:49) and natural indoor temperature (19–21°C). All the horses were feed traditional rations, based hay and a mix of cereals (oats and barley), it was provided three times daily (at 08:00, 12:00 and 17:00). Water was available ad libitum. The horses were trained from 15:00 to 16:00. Training included walking, trotting, galloping and obstacle jumping.

Blood samples were collected at four-hour intervals over a 48-hour period (starting at 08:00 hours on day 1 and finishing at 4:00 on day 2) via an intravenous cannula inserted into the jugular vein. Blood samples were immediately centrifuged for 10 min at 3000 rpm with a standardized procedure and stored at + 4°C for a maximum of 24 h. Individual serum samples were deproteinized with 5-sulfosalicylic acid (5-SSA), centrifuged for 10 min at 3000 rpm, and immediately processed. On the filtered supernatant, the concentrations of tryptophan and serotonin were assessed by high-performance liquid chromatography (HPLC).

All the results were expressed as mean ± SD. One-way repeated measures analysis of variance (ANOVA) was used to determine significant difference. Probabilities < 0.05 were considered statistically significant. In addition, we applied a trigonometric statistical model to the average values of each time series, so as to describe the periodic phenomenon analytically, by individuating the main rhythmic parameters according to the single cosinor procedure [13]: Mesor (Midline Estimating Statistic of Rhythm), expressed in the same conventional unit of the relative parameter, with the confidence interval (C.I.) at 95%, Amplitude (A), expressed in the same unit as the relative Mesor, and Acrophase (Φ), expressed in hours with 95% confidence intervals.

## Results

**Figure 1:**
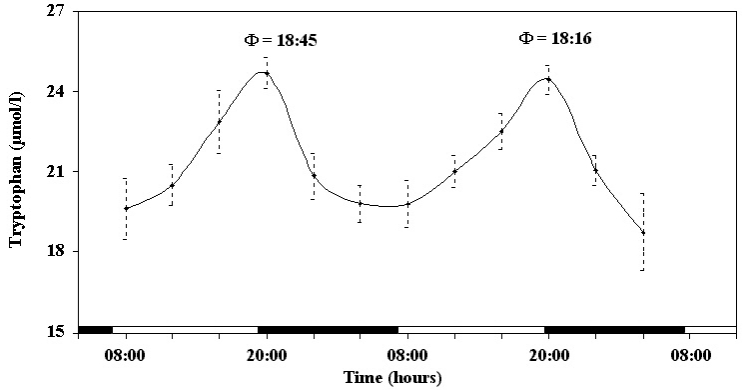
**Daily rhythm of tryptophan blood level in the horse**. Each time point represents the mean value ± SD. Φ represents the acrophase. Black and white stripes at the bottom of the graphic represent dark and light duration of the natural photoperiod.

**Figure 2:**
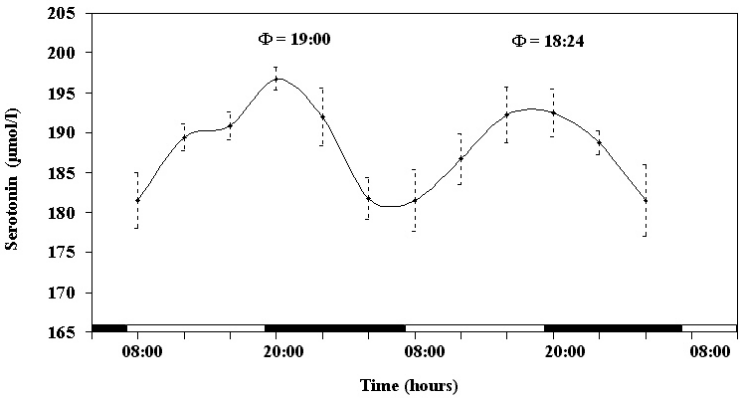
**Daily rhythm of serotonin blood level in the horse**. Each time point represents the mean value ± SD. Φ represents the acrophase. Black and white stripes at the bottom of the graphic represent dark and light duration of the natural photoperiod.

The results obtained during the experimental period indicate the existence of daily rhythms of tryptophan and serotonin serum concentration in the horse, as shown in Figures 1 and 2. ANOVA showed a highly significant effect of time on serum concentration of tryptophan and serotonin, in either day, as follows: tryptophan F_(11,44) _= 38.41, p < 0.0001; serotonin F_(11,44) _= 64.21, p < 0.0001. The application of the periodic model and the statistical analysis of the cosinor procedure enabled us to define the periodic parameters and their acrophases (expressed in hours) during the 2 days of monitoring. Both parameters studied showed nocturnal acrophases, as follows: tryptophan at 18:45 in the first day and at 18:16 in the second day; serotonin at 19:00 in the first day and 18:24 in the second day.

## Conclusion

The results obtained in this study outline a nycthemeral pattern regarding blood levels of serotonin and tryptophan. For both parameters, acrophases occurred during the evening hours at the onset of the dark phase of the experimental dark/light cycle. This suggest that photoperiod affects the timing of the investigated parameters since animals exposed to an autumnal photoperiod showed nocturnal acrophase (tryptophan at 00:40 and serotonin at 00:28) [1].

Tryptophan acrophase occurred 30 minutes earlier than serotonin acrophase. This is consistent with the role of tripthophan hydroxilation in the control of 5-HT biosynthesis. However, various patterns of daily variation of 5-HT and 5-hydroxyindoleacetic acid (5-HIAA) were observed in rats, suggesting that the nycthemeral factors affecting serotonin metabolism can be different among brain areas [16].

It has long been known that nutritional status can alter brain neurochemistry, especially that involving carbohydrates and serotonin [5,8,19]. It has been hypothesized that tryptophan infusion may increase fTrp (free-tryptophan) and the fTrp-to-BCAA (branched-chain amino acids) ratio in plasma at the same time as it decreases treadmill endurance in horses [7]. Thus central fatigue may limit endurance capacity in horses and, by manipulating fTrp and BCAA, equine exercise capacity might be altered predictably [7]. Tryptophan infusion results are consistent with the central fatigue hypothesis that an increased plasma fTrp concentration is related to the early onset of fatigue during prolonged exercise [15]. Therefore, it is likely that exercise performed at the time of the acrophase of the tryptophan rhythm (18:45, 18:16) affects the onset of physiological fatigue, thus turning on the body's exercise adaptation mechanisms in order to maintain better physical performance.

## Authors' contributions

GP- Designed the study and conducted statistical analysis.

AA- Conducted bibliographic research.

FF- Carried out the data collection procedure.

MP- Carried out the data collection procedure.

GC- Supervised the data collection procedures and conducted bibliographic research.

All authors read and approved the final manuscript.
